# Task-Oriented Deep Joint Source-Channel Coding with Semantic-Aware Adaptive Quantization for Autonomous Driving

**DOI:** 10.3390/s26165035

**Published:** 2026-08-08

**Authors:** Xin Wang, Haiqiang Chen, Youming Sun, Xiangcheng Li, Min Xie

**Affiliations:** Guangxi Key Laboratory of Multimedia Communications and Network Technology, Guangxi University, Nanning 530004, China; 2413392025@st.gxu.edu.cn (X.W.); haiqiang@gxu.edu.cn (H.C.); ymsun@gxu.edu.cn (Y.S.); xcli@gxu.edu.cn (X.L.)

**Keywords:** semantic communication, deep joint source-channel coding, autonomous driving, semantic segmentation, adaptive quantization, channel bandwidth ratio

## Abstract

In autonomous driving and intelligent transportation, vehicles need to share visual perception information to extend sensing range, but conventional separate source-channel coding can suffer from the cliff effect under harsh vehicular channels, causing downstream perception failures. This paper proposes an end-to-end semantic communication system for autonomous-driving semantic segmentation. The system adopts SegFormer-B2 as the semantic encoder, performs feature modulation conditioned on the signal-to-noise ratio SNR, applies semantic-aware adaptive bit-width quantization based on Gumbel-Softmax, and uses a task-oriented decoder to directly produce segmentation maps. Experiments on Cityscapes evaluate the system under additive white Gaussian noise (AWGN) and per-channel independent block Rayleigh fading channels, with comparisons against a JPEG-based separate baseline and fixed-bit-width variants. Under AWGN, the proposed system maintains a mean intersection over union above 0.70 at SNR=−6dB, while the conventional baseline fails at SNR=0dB with an mIoU of 0.02, indicating improved robustness against the cliff effect. The results also show a non-monotonic relationship between quantization precision and segmentation performance: fixed 4-bit quantization can outperform fixed 6-bit quantization because finer quantization is more sensitive to channel noise. Under Rayleigh fading, adaptive quantization may suffer from semantic-channel mismatch, providing useful observations for future channel-aware semantic resource allocation.

## 1. Introduction

With the growing demand for autonomous driving and intelligent transportation systems [1,2], autonomous vehicles increasingly require high-precision environmental perception to ensure driving safety. However, a single vehicle is inherently limited by occlusion, blind spots, and the finite sensing range of onboard sensors. Sharing visual perception data through vehicle-to-everything (V2X) networks is an important approach to extending the perception range [3]. Although V2X can improve the perceptual coverage of driving scenarios, visual data in vehicular networks are typically large in volume, making them difficult to transmit over limited wireless spectrum resources. Conventional transmission schemes therefore face severe challenges.

Traditional wireless image transmission follows the separate source-channel coding paradigm. However, this separate architecture may suffer a sharp performance collapse under poor channel conditions, commonly referred to as the cliff effect [4]. Unfavorable channel conditions are unavoidable in practical vehicular transmission scenarios. High vehicle mobility and multipath fading can cause rapid channel degradation, making the cliff effect particularly pronounced. In such cases, the effective communication link of a conventional scheme may fail completely, preventing the transmission of visual data required by downstream perception tasks. Therefore, a new transmission paradigm is needed to address this issue.

In recent years, deep joint source-channel coding (DeepJSCC) has attracted attention as a new paradigm [4,5,6,7,8]. Through end-to-end joint optimization, it can mitigate the cliff effect of conventional separate schemes under low-SNR conditions. At the same time, semantic communication has been widely studied as a communication paradigm beyond bit-level reliability [9,10]. Based on task-oriented semantic communication, existing works have further applied this idea to image segmentation tasks [11,12]. However, current semantic communication studies for segmentation mainly focus on encoder architectures and loss function design. The feature quantization mechanism, namely how semantic features should be discretized for transmission and how this process interacts with channel robustness, has not yet been systematically studied. From the perspective of V2X perception sharing, the practical objective is not to reconstruct visually faithful images, but to maintain usable downstream segmentation outputs under limited bandwidth and harsh channel conditions. Preserving segmentation of road regions, vehicles, pedestrians, and traffic facilities can help reduce perception interruption when conventional image bitstreams fail, although this paper does not claim to validate a complete deployed autonomous-driving safety system.

To address the above issues, this paper proposes an end-to-end semantic communication system for autonomous-driving semantic segmentation. The system adopts SegFormer-B2 as the semantic encoding backbone [13], introduces an SNR-conditioned feature modulation module, incorporates a semantic-aware adaptive bit-width quantization mechanism, and employs a task-oriented decoder at the receiver to directly output segmentation results. The system is evaluated under both AWGN and Rayleigh fading channels, with a focus on the relationship among quantization precision, channel conditions, and segmentation performance.

The main contributions of this paper are summarized as follows.

(1) End-to-end semantic communication system design: We propose an end-to-end DeepJSCC system for autonomous-driving semantic segmentation. The system integrates semantic encoding, channel adaptation, adaptive quantization, and task-oriented decoding. The system mitigates the cliff effect of the conventional separate scheme under both AWGN and Rayleigh channels and exhibits graceful degradation.
(2) Design and limitation analysis of the channel-conditioned modulation module: We design a lightweight SNR-driven feature modulation module, referred to as ChannelAdapter, and analyze its actual role under different channels through ablation experiments. The results show that this module has only a minor impact under AWGN and has not yet produced stable gains under Rayleigh fading. This indicates that simple SNR-conditioned feature modulation is still insufficient to resolve the semantic-channel mismatch problem under fading channels.
(3) Semantic-aware adaptive quantization mechanism: We propose a two-dimensional semantic utility evaluation method based on information entropy and class importance, and combine it with Gumbel-Softmax [14] to enable end-to-end learnable non-uniform bit-width allocation.
(4) Systematic analysis of the relationship between quantization precision and channel robustness: Through controlled experiments, we reveal a non-monotonic trade-off between quantization precision and channel robustness. A higher quantization precision, such as 6-bit, performs worse than the medium-precision 4-bit setting under noisy channels. We also provide a restrained analysis of the limitations of adaptive quantization under fading channels. This finding provides useful references for future quantization design in semantic communication systems.

## 2. Related Work

### 2.1. Deep Joint Source-Channel Coding

In recent years, deep joint source-channel coding (DeepJSCC) has attracted broad attention as a new wireless transmission paradigm. Bourtsoulatze et al. [4] first proposed an end-to-end DeepJSCC architecture based on convolutional autoencoders, which directly maps image pixels to complex-valued channel symbols. Their experiments showed that this approach eliminates the cliff effect observed in conventional separate source-channel coding schemes during decoding under low-SNR conditions, thereby establishing a core paradigm for subsequent semantic communication research.

To further improve the adaptability of DeepJSCC under dynamic channel conditions, Xu et al. [5] proposed ADJSCC, namely attention-based deep learning JSCC, which embeds the channel SNR as an auxiliary input into the encoder and decoder so that a single model can cover a wide range of channel conditions. This work provides a basic design pattern for later channel-aware DeepJSCC methods. Following a similar idea, Ding et al. [6] proposed an SNR-adaptive variable-rate JSCC scheme. In addition, Kurka and Gündüz [8] proposed DeepJSCC-f, further exploring the use of channel output feedback in end-to-end image transmission.

Regarding quantization mechanisms, Tung et al. [7] proposed DeepJSCC-Q, which systematically introduces constellation-constrained quantization into the DeepJSCC framework and studies end-to-end joint optimization under discrete channel-symbol constraints. This work treats quantization as an independent dimension in DeepJSCC research and provides an important basis for semantic communication systems compatible with digital modulation.

It should be noted that the above studies mainly target image or video reconstruction quality, using distortion metrics such as PSNR and MS-SSIM as optimization objectives. Their encoders learn general visual representations for reconstruction. When the downstream objective shifts to a specific perception task, such as segmentation, detection, or classification, the system design principles need to be reconsidered. This forms another major direction in semantic communication research.

### 2.2. Task-Oriented Semantic Communication

Unlike classical communication systems that aim to recover bits or symbols, task-oriented semantic communication directly optimizes the performance of downstream tasks. In the vision of semantic communication for 6G networks, Strinati and Barbarossa [15] explicitly argued that future communication systems should move beyond the Shannon paradigm and incorporate “semantics” and “task effectiveness” into their design. Shao et al. [16] further formulated task-oriented communication as an information bottleneck optimization problem, using the trade-off between rate, distortion, and relevance to guide joint feature extraction and transmission. This provides a systematic theoretical framework for the field.

At the application level, Xie et al. [17] proposed DeepSC, which applies the Transformer architecture to text semantic communication and evaluates system performance using sentence-level semantic similarity rather than bit error rate. It shows advantages over conventional schemes under low-SNR conditions. For video transmission, Wang et al. [18] proposed DVST, which realizes end-to-end wireless video semantic transmission through temporally adaptive encoding. Together, these works establish the basic design principle of task-oriented semantic communication: under bandwidth and channel constraints, the system objective should be directly aligned with the effectiveness metric of the downstream task, rather than the fidelity of an intermediate representation.

### 2.3. Semantic Communication for Visual Perception in Vehicular Scenarios

Applying semantic communication principles to vehicular visual perception tasks has become an active research direction in recent years. Existing studies mainly focus on two aspects: encoder architecture design and task loss function design. In terms of encoder architecture, Yang et al. [19] proposed WITT, a wireless image transmission Transformer, showing that Transformer backbones can outperform CNN architectures in wireless image transmission. Pan et al. [11] further introduced the multi-scale feature extraction capability of Swin Transformer into a semantic communication system for semantic segmentation, using window-based self-attention to achieve efficient semantic encoding of vehicular images.

In terms of task loss design, considering the differences in safety importance among object categories in autonomous-driving scenarios, Lyu et al. [12] proposed VIS-SemCom. By introducing an importance-aware loss and online hard example mining (OHEM), it improves the segmentation accuracy of critical categories such as pedestrians and vehicles. This work also considers vehicular channel models involving Doppler effects and shadow fading. Chen et al. [20] proposed an importance-aware loss for autonomous-driving semantic segmentation, emphasizing safety-critical categories such as vehicles, pedestrians, and traffic signs through class-importance weighting. This provides a visual-task-level reference for the class-importance modeling used in this paper. In addition, Ye et al. [21] provided a systematic survey of semantic communication in the Internet of Vehicles, covering key techniques from semantic information extraction and communication architecture to resource management, thereby offering a comprehensive overview of the field. Feng et al. [22] further discussed semantic communication for edge-intelligence-enabled autonomous driving, highlighting multi-user collaboration and multi-task execution as important deployment challenges.

Overall, existing semantic communication studies for vehicular visual perception mainly improve system performance from the perspectives of network architecture and task loss function design. However, two limitations remain. First, most segmentation-oriented works do not explicitly analyze how discretizing semantic features affects channel robustness. Second, existing channel-aware designs usually treat the channel state as a conditioning signal for the neural codec, but their interaction with task-oriented quantization under fading channels remains insufficiently understood. As discussed in Section 2.1, works such as DeepJSCC-Q have demonstrated the importance of quantization mechanisms in image reconstruction tasks, but the corresponding research has not yet been sufficiently extended to segmentation-oriented semantic communication scenarios. To address this gap, this work designs a semantic-aware adaptive quantization mechanism and a channel-conditioned feature modulation module for segmentation-oriented semantic communication, and systematically examines the interactions among quantization precision, channel conditions, and segmentation performance.

## 3. System Model

### 3.1. System Architecture

**Figure 1:**
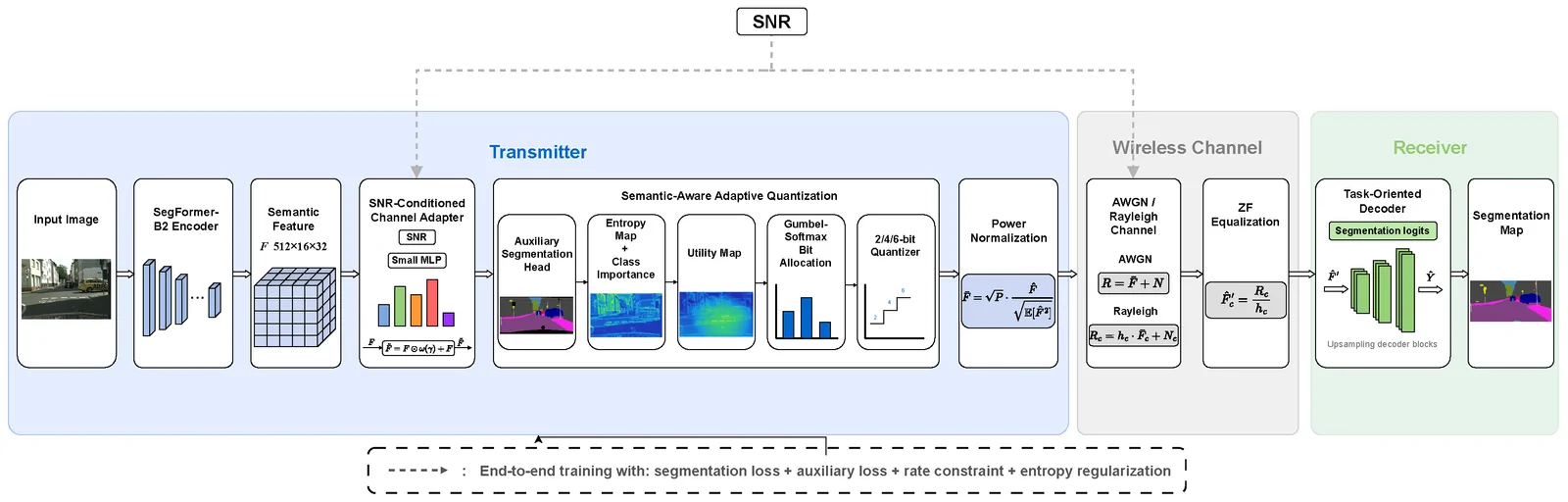
System architecture of the proposed semantic communication framework for autonomous-driving semantic segmentation.

The end-to-end semantic communication system proposed in this paper is designed for real-time semantic segmentation in autonomous-driving scenarios. The overall architecture is shown in Figure 1. The transmitter consists of a semantic encoder, a channel adapter, and a semantic-aware adaptive quantizer, while the receiver consists of a wireless channel equalization module and a semantic decoder. The entire system is optimized through end-to-end joint training. In this architecture, the SegFormer-B2 encoder and the channel/equalization models are adopted as established components, whereas the main newly designed part is the semantic-aware adaptive quantization module; the ChannelAdapter is retained as a lightweight diagnostic modulation block whose limited standalone gains are analyzed in Section 5.4. This paper focuses on a single-link feature transmission setting in order to isolate the interaction among semantic feature extraction, quantization, and channel perturbation. Multi-user access, scheduling, pilot collision, and inter-user interference are beyond the current experimental scope; related collision-resilient random-access designs [23] provide useful references for future multi-user extensions.

The data flow is as follows. The input image is first processed by the semantic encoder to extract high-level semantic features. The features are then fed into the channel adapter, which recalibrates the channel dimension according to the current signal-to-noise ratio SNR. The adapted features are passed through the adaptive quantization module for spatially non-uniform bit-width allocation. The quantized features are then transmitted over the wireless channel as baseband-equivalent channel symbols. At the receiver, equalization is performed under the assumption of perfect channel state information (CSI). Finally, the semantic decoder directly maps the perturbed features to pixel-level class predictions, without reconstructing an intermediate image.

### 3.2. Signal Model and Notation

For clarity, this section summarizes the main notation used throughout the paper. Let the input image be $X\in R^{3\times H\times W}$, where H=512 and W=1024 denote the image height and width, respectively. The corresponding ground-truth semantic segmentation label is denoted by $Y\in {\left\{1,\ldots ,K\right\}}^{H\times W}$ and K=19 denotes the number of semantic classes in the Cityscapes dataset.

The semantic encoder $f_{enc}\left(\cdot ;\theta_{enc}\right)$ maps *X* to a semantic feature tensor $F\in R^{C\times H^{\prime }\times W^{\prime }}$, where C=512, $H^{\prime }=16$, and $W^{\prime }=32$. To avoid ambiguity among the intermediate feature variables, this paper uses the following notation. The encoder output is denoted by *F*. After channel-adaptive modulation, the feature becomes $\tilde{F}$. After adaptive quantization, it becomes $\hat{F}=Q\left(\tilde{F}\right)$. After per-sample power normalization, the actually transmitted feature is denoted by $\bar{F}$. The channel observation is denoted by *R*, and the equalized receiver-side feature is denoted by ${\hat{F}}^{\prime }$. Therefore, $\hat{F}$ refers to the quantized pre-channel feature, whereas ${\hat{F}}^{\prime }$ refers to the feature available to the semantic decoder after channel transmission and receiver-side equalization. The wireless channel and equalization are represented by the operator $H_{\gamma }\left(\cdot \right)$, where γ denotes the SNR in dB. The semantic decoder $g_{dec}\left(\cdot ;\theta_{dec}\right)$ maps ${\hat{F}}^{\prime }$ to segmentation logits, which are then bilinearly upsampled and processed by pixel-wise argmax to obtain the final segmentation prediction $\hat{Y}$.

The entire end-to-end system can be written in stages as follows:

(1) $$\begin{array}{cccc} F & =f_{enc}\left(X;\theta_{enc}\right),\; & \; & (\mathrm{semantic}\;\mathrm{encoding}) \end{array}$$

(2) $$\begin{array}{cccc} \tilde{F} & =\mathrm{Adapt}(F,\gamma ),\; & \; & (\mathrm{channel}\;\mathrm{adaptation}) \end{array}$$

(3) $$\begin{array}{cccc} \hat{F} & =Q(\tilde{F}),\; & \; & (\mathrm{adaptive}\;\mathrm{quantization}) \end{array}$$

(4) $$\begin{array}{cccc} \bar{F} & =\mathrm{Norm}(\hat{F}),\; & \; & (\mathrm{power}\;\mathrm{normalization}) \end{array}$$

(5) $$\begin{array}{cccc} {\hat{F}}^{\prime } & =H_{\gamma }\left(\bar{F}\right),\; & \; & (\mathrm{channel}\;\mathrm{transmission}\;\mathrm{and}\;\mathrm{equalization}) \end{array}$$

(6) $$\begin{array}{cccc} \hat{Y} & =g_{dec}\left({\hat{F}}^{\prime };\theta_{dec}\right),\; & \; & (\mathrm{semantic}\;\mathrm{decoding}) \end{array}$$

where Adapt(·,γ) denotes the channel adapter defined in Section 4.2, Q(·) denotes the semantic-aware adaptive quantization operator defined in Section 4.3, Norm(·) denotes the power-normalization operation in Section 4.4, and $H_{\gamma }\left(\cdot \right)$ denotes the wireless channel and receiver-side equalization operator defined in Section 4.4.

### 3.3. Problem Formulation

Unlike conventional separate source-channel coding, which aims to minimize end-to-end distortion, this paper adopts a task-oriented optimization paradigm: the direct objective of the system is the performance of the segmentation task, rather than the reconstruction fidelity of an intermediate representation.

Formally, given a bandwidth constraint and channel conditions, the optimization problem can be formulated as:

(7) $$\min_{\theta_{enc},\theta_{adapt},\theta_{Q},\theta_{dec}}E_{X,Y,\gamma }\left[L_{seg}\left(\hat{Y},Y\right)\right]\;s.t.\;E\left[\bar{b}\right]\leq b_{target}.$$

where $L_{seg}$ is the pixel-wise cross-entropy loss, $\bar{b}$ is the average latent bit-width of the system, defined as the average assigned bit-width over all spatial feature locations, and $b_{target}=4.0$ is the target bit-width budget. The expectation is taken over the training data distribution, the channel condition distribution, and the randomly sampled SNR distribution during training.

The bandwidth constraint is softly introduced during training through the rate constraint loss described in Section 4.6.

## 4. Proposed Method

### 4.1. Semantic Encoder

To build an end-to-end communication system for semantic segmentation, the encoder needs to compress source redundancy while preserving high-level semantic representations that are most discriminative for the downstream pixel-level classification task. Considering the strong performance of the SegFormer family on autonomous-driving semantic segmentation benchmarks such as Cityscapes [24], as well as its hierarchical Transformer structure for modeling multi-scale semantic features, this paper adopts the MiT-B2 encoder of SegFormer-B2 [13] as the semantic feature extraction backbone.

Specifically, after the input image $X\in R^{3\times H\times W}$ (H=512, W=1024) passes through the four-stage hierarchical encoder, this paper selects the feature map from the fourth stage, namely the deepest stage, as the semantic representation to be transmitted:

(8) $$F=f_{enc}\left(X;\theta_{enc}\right)\in R^{C\times H^{\prime }\times W^{\prime }}.$$

where C=512 is the number of channels, $H^{\prime }=16$, and $W^{\prime }=32$ denote the spatial size after downsampling to 1/32 of the original image resolution, and $\theta_{enc}$ denotes the encoder parameters. The fourth-stage output is selected instead of lower-level features because deep features have aggregated semantic information over a larger receptive field and contain lower spatial redundancy, making them suitable as transmission carriers over bandwidth-limited channels. At the same time, their level of semantic abstraction is closely aligned with the downstream segmentation task, which can reduce the optimization difficulty in end-to-end training.

To preserve the general semantic priors learned by the pretrained SegFormer model on ImageNet and Cityscapes, this paper uses a relatively low learning rate for the encoder parameters during end-to-end training, denoted as $6\times 10^{-5}$, which is much lower than the learning rate used for the other system modules, denoted as $3\times 10^{-4}$. This layer-wise learning-rate strategy prevents gradient noise introduced by the wireless channel from destructively updating the pretrained parameters, while still allowing the encoder to perform limited fine-tuning toward the feature distribution required by the communication scenario.

### 4.2. Channel-Adaptive Feature Enhancement

In practical wireless transmission scenarios, channel conditions vary over time and space, whereas the semantic features *F* produced by the encoder are static representations that are independent of the channel state. If *F* is directly transmitted over the channel, the system may face two types of mismatch: under high-SNR conditions, it may fail to fully exploit the available channel capacity; under low-SNR conditions, the features may be sensitive to noise and therefore suffer sharp performance degradation. To this end, this paper designs an SNR-conditioned feature modulation module, referred to as the channel adapter [25], which attempts to conditionally recalibrate semantic features according to the current channel state.

Specifically, given the current SNR γ (dB), the channel adapter maps it to a channel-wise gating vector through a lightweight two-layer multilayer perceptron (MLP):

(9) $$\omega =\sigma \left(W_{2}\cdot \mathrm{ReLU}\left(W_{1}\cdot \tilde{\gamma }\right)\right)\in R^{C}.$$

where $\tilde{\gamma }=\left(\gamma -6\right)/12$ is the normalized SNR, $W_{1}\in R^{(C/4)\times 1}$ and $W_{2}\in R^{C\times (C/4)}$ are learnable parameters of the MLP, ReLU(·) denotes the rectified linear unit activation, and σ(·) denotes the sigmoid activation function. The normalization maps the training SNR range [−6,18] dB to approximately [−1,1]. The matrices $W_{1}$ and $W_{2}$ are initialized randomly and optimized jointly with the other trainable modules through backpropagation; they are not manually selected. The gating vector ω is broadcast along the spatial dimensions, multiplied with the original feature in a channel-wise manner, and then added back to the original feature through a residual connection:

(10) $$\tilde{F}=F⊙\omega +F.$$

where ⊙ denotes channel-wise multiplication. The residual connection ensures that, when the adapter is initialized or fails to provide useful modulation, the system can still degrade to a no-adapter baseline behavior, thereby improving training stability.

It should be clarified that this module is fundamentally different from conventional channel attention mechanisms, such as Squeeze-and-Excitation [26]. In the latter, gating weights are computed from the feature map itself through global pooling and reflect inter-channel correlations within the feature representation. In contrast, the gating signal in the proposed module is entirely driven by external channel state information, namely SNR, and does not depend on the content of *F*. This design positions the module explicitly as a channel-condition-aware mechanism rather than a general feature-saliency modeling module. Under the multi-SNR joint training strategy described in Section 4.6 [5], this module learns an implicit mapping between SNR and channel weights through backpropagation, thereby providing a trainable implementation of channel-state-aware feature modulation.

### 4.3. Semantic-Aware Adaptive Quantization

In the end-to-end semantic communication system, semantic features need to be quantized before being transmitted over the wireless channel. The choice of quantization precision involves a trade-off between fidelity and physical robustness. Higher quantization precision can preserve richer semantic details, but the finer quantization intervals make the retained numerical differences more susceptible to channel noise, which may cause cross-level quantization shifts. Lower quantization precision is naturally more robust to noise, but it may lead to severe semantic information loss.

Conventional fixed quantization applies a single uniform quantization precision to all spatial locations, and therefore fails to exploit the strong spatial semantic non-uniformity in autonomous-driving scenes. For example, object boundaries and small objects, such as pedestrians and traffic lights, are highly semantically sensitive, whereas large homogeneous regions, such as sky and road areas, are semantically redundant. Based on this observation, this paper defines three discrete quantization levels, namely 2-bit for low precision, 4-bit for medium precision, and 6-bit for high precision, and proposes an adaptive allocation mechanism based on spatial utility awareness.

#### 4.3.1. Two-Dimensional Semantic Importance Estimation

To quantify the semantic value of spatial features, we attach a lightweight auxiliary segmentation head to the downsampled encoder output, whose spatial resolution is 1/32 of the original image. This head outputs a 19-class probability distribution $p^{\left(i,j\right)}$ at each spatial location (i,j). Two metrics are then computed.

(a) **Information entropy metric:** H. The normalized information entropy $H\left(i,j\right)=-\frac{1}{\log(19)}\sum_{c=1}^{19}p_{c}\left(i,j\right)\log\left(p_{c}\left(i,j\right)\right)$ is computed at each spatial location. A high entropy value indicates severe class uncertainty in that region, such as at the boundary between multiple semantic classes, and therefore suggests that higher precision may be needed to preserve transitional details.
(b) **Class importance metric:** I. A learnable class-value vector v$\in R^{19}$ is introduced to automatically learn the importance weight of each class through end-to-end training. The notation v is used here to distinguish the class-value vector from the channel-adapter gating vector ω in Section 4.2. For each spatial location, the class with the maximum predicted probability from the auxiliary segmentation head $c^{*}=\arg\;\max_{c}p_{c}\left(i,j\right)$ is selected, and the corresponding learnable weight is queried. In implementation, the unconstrained class weights are passed through a softplus activation to keep the effective weights non-negative. The importance values of all spatial locations within the same image are then min-max normalized to [0,1], yielding the importance score I(i,j) for that location. Since this estimation is performed on a low-resolution feature map, this metric provides a coarse regional semantic tendency rather than pixel-level segmentation.

The two metrics are fused through learnable parameters α and β to obtain the final semantic utility map:

(11) U(i,j)=α·H(i,j)+β·I(i,j).

The fusion parameters α and β are also learned during end-to-end training. In implementation, they are parameterized through softplus activations, so that the effective fusion weights remain non-negative while their relative magnitudes are determined by the segmentation and rate-control objectives.

#### 4.3.2. Probability Generation and Normalized Mapping

To map the continuous utility value *U* to discrete allocation probabilities, this paper introduces two learnable decision thresholds, $t_{low}$ and $t_{high}$, with softplus activation used to ensure $t_{high}>t_{low}>0$. In implementation, $t_{low}$ is obtained from a learnable raw parameter through softplus, and $t_{high}$ is obtained by adding a positive learned gap to $t_{low}$. The sharpness parameter *s* is also learned through a softplus parameterization. These parameters are initialized to provide a smooth low/medium/high split and are subsequently selected by end-to-end optimization rather than manual tuning. Based on the sigmoid function, the initial preferences for assigning the highest and lowest precision levels are computed, while the preference for the medium precision level is obtained through a residual truncation mechanism:

(12) $$\begin{array}{cc} p_{high} & =\sigma \left(s\cdot (U-t_{high})\right),\\ p_{low} & =\sigma \left(s\cdot (t_{low}-U)\right),\\ p_{mid} & =\max\left(1-p_{high}-p_{low},\varepsilon \right). \end{array}$$

where $\varepsilon =10^{-6}$ is a small numerical constant used to prevent probability underflow. The value of ε is not treated as a tunable hyperparameter; other sufficiently small values would have the same practical role of avoiding zero probabilities. The quantities $p_{low}$, $p_{mid}$, and $p_{high}$ are unnormalized allocation preferences. They correspond to the 2-bit, 4-bit, and 6-bit levels, respectively, i.e., $p_{2}=p_{low}$, $p_{4}=p_{mid}$, and $p_{6}=p_{high}$. These preferences are then normalized at each spatial location to obtain the final soft allocation probability for each quantization level k∈{2,4,6}:

(13) $$\pi_{k}=\frac{p_{k}}{p_{high}+p_{low}+p_{mid}+\varepsilon }.$$

During training, a rate constraint loss is used to control the global average quantization bit-width so that it stays close to the target value. The target average latent bit-width is set to 4.0, enabling fair comparison with the fixed 4-bit baseline under the same average quantization bit-width.

#### 4.3.3. Differentiable Discretization Training: Hard Gumbel-Softmax

Discrete quantization-level selection contains a non-differentiable hard decision operation, which would block backpropagation through the end-to-end system if applied directly. This paper introduces the Hard Gumbel-Softmax technique to address this gradient bottleneck [14]. We feed the logits of the normalized probabilities $\log(\pi_{k})$ into the Gumbel-Softmax module. During training, the system performs a hard decision and directly outputs the quantization level with the maximum probability, while gradients are passed through the straight-through estimator [27]:

(14) $$Q_{\mathrm{choice}}=\mathrm{one-hot}\left(\arg\max_{k\in \{2,4,6\}}\frac{\log\left(\pi_{k}\right)+G_{k}}{\tau }\right).$$

where $G_{k}$ denotes independent noise sampled from the Gumbel(0,1) distribution, which provides exploration during the early training stage. The one-hot(·) operator converts the selected bit-width index into a three-dimensional binary allocation vector, where only one of the 2-bit, 4-bit, and 6-bit entries is active at each spatial location. During backpropagation, gradients are computed along the continuous Softmax relaxation path. The temperature parameter τ controls the smoothness of the relaxed distribution. It follows an exponential annealing schedule during training, decaying from 5.0 to 0.5, so that the bit-width allocation has stronger stochastic exploration in the early stage and gradually approaches deterministic hard decisions as training converges.

During inference, the system no longer injects Gumbel noise and directly applies argmax to the normalized probabilities for deterministic allocation.

### 4.4. Wireless Channel Model

After the output of the quantization module described in Section 4.3 is obtained, the quantized semantic feature $\hat{F}\in R^{C\times H^{\prime }\times W^{\prime }}$ is used as the input to the physical channel module. It should be emphasized that $\hat{F}$ is a pre-channel feature. The actually transmitted baseband-equivalent symbols are the power-normalized feature $\bar{F}$. To satisfy a typical transmit power constraint, this paper first applies per-sample power normalization to $\hat{F}$:

(15) $$\bar{F}=\sqrt{P}\cdot \frac{\hat{F}}{\sqrt{E[{\hat{F}}^{2}]}}.$$

where *P* denotes the target average symbol power, set to P=1 in this work, and $E[{\hat{F}}^{2}]$ denotes the average squared value over all elements of each feature sample. Setting P=1 is a standard unit-power normalization convention: it fixes the transmitted symbol power scale, while the SNR γ controls the corresponding noise variance. With P=1, this equation is exactly the per-sample normalization used in implementation, i.e., division by the square root of the mean squared feature value.

This paper considers two typical channel models. The first is the additive white Gaussian noise (AWGN) channel, whose input–output relationship is given by:

(16) $$R=\bar{F}+N,\;N∼N\left(0,\sigma_{n}^{2}\right).$$

where the noise variance $\sigma_{n}^{2}=P/10^{\gamma /10}$ is determined by the current SNR.

The second is the Rayleigh fading channel. Since different channels of the semantic feature are treated as independent transmission resources in this paper, we adopt a per-channel independent block fading model: each feature channel c∈{1,…,C} experiences an independent Rayleigh-distributed fading amplitude during one transmission interval, and the fading coefficient remains constant across all spatial locations within that channel. Specifically:

(17) $$R_{c}=h_{c}\cdot {\bar{F}}_{c}+N_{c},\;h_{c}=\left|g_{c}\right|,\;g_{c}∼\mathrm{CN}\left(0,1\right),\;N_{c}∼N\left(0,\sigma_{n}^{2}\right),\;c=1,\ldots ,C.$$

where ${\bar{F}}_{c}$ denotes the *c*-th channel slice of the power-normalized transmitted feature $\bar{F}$, $g_{c}$ is an auxiliary complex Gaussian random variable used to generate the Rayleigh amplitude $h_{c}$, and $N_{c}$ denotes the additive real-valued Gaussian noise on the corresponding subchannel. This model corresponds to a transmission scheme that maps semantic feature channels to independently fading subchannels, and can be regarded as a reasonable abstraction of a narrowband block fading scenario. At the receiver, zero-forcing (ZF) equalization is performed under the assumption of perfect channel state information (CSI):

(18) $${\hat{F}}_{c}^{\prime }=\frac{R_{c}}{h_{c}}={\bar{F}}_{c}+\frac{N_{c}}{h_{c}}.$$

It should be noted that ZF equalization can strongly amplify the effective noise when $\left|h_{c}\right|$ is small, which is an important reason why system performance is more prone to degradation under the Rayleigh channel. Designing an effective channel-adaptive mechanism for this fading characteristic is the direction that the channel adapter in Section 4.2 attempts to explore.

In implementation, the channel is simulated in a real-valued baseband-equivalent form, where the Rayleigh fading coefficient is represented by the fading amplitude $h_{c}=\left|g_{c}\right|$ of a complex Gaussian channel. This simplification is commonly used in DeepJSCC-style simulations and does not affect the qualitative analysis of fading-induced robustness degradation.

### 4.5. Semantic Decoder

Unlike conventional communication systems that first reconstruct the image and then perform the downstream task in a cascaded manner, this paper adopts a task-oriented end-to-end design [16]. The receiver-side decoder directly maps the channel-perturbed semantic features to pixel-level class predictions, without reconstructing an intermediate image. The rationale is that, since the downstream task is semantic segmentation, the transmission bandwidth and channel resources consumed by image reconstruction do not directly contribute to the final task and may instead introduce unnecessary conflicts in the optimization objective.

Specifically, the semantic decoder $g_{dec}\left(\cdot ;\theta_{dec}\right)$ consists of several lightweight upsampling convolutional blocks, which progressively restore the equalized receiver-side features ${\hat{F}}^{\prime }\in R^{C\times H^{\prime }\times W^{\prime }}$ to segmentation logits at 1/4 of the original image resolution:

(19) $$L=g_{dec}\left({\hat{F}}^{\prime };\theta_{dec}\right)\in R^{K\times H/4\times W/4}.$$

where K=19 is the number of semantic classes in the Cityscapes dataset. The final segmentation prediction is obtained by bilinearly upsampling *L* to the original image resolution and then applying pixel-wise argmax.

The decoder is intentionally kept lightweight so that the computational cost of the system is mainly concentrated in the semantic extraction stage of the encoder, which is consistent with the deployment constraints of resource-limited vehicular terminals. During end-to-end training, the decoder needs to adapt jointly to three sources of perturbation: lossy quantization, AWGN noise, and Rayleigh fading. This joint optimization is key to maintaining robustness under low-SNR conditions.

### 4.6. Joint Training Strategy

To enable the system to maintain stable performance over a wide range of SNR conditions, this paper trains separate models for the AWGN and Rayleigh channels. During the training of each model, the SNR is uniformly sampled from the training set $S_{\mathrm{train}}=\left\{-6,0,6,12,18\right\}\;\mathrm{dB}$. This multi-SNR training strategy forces the encoder, channel adapter, and decoder to jointly adapt to SNR conditions spanning a 24 dB range, thereby avoiding the overfitting problem caused by training at a fixed SNR.

The composite loss function of the system consists of four terms:

(20) $$L=L_{seg}+\lambda_{aux}\cdot L_{aux}+\lambda_{rate}\cdot L_{rate}+\lambda_{ent}\cdot L_{ent}.$$

where:

(a) The main segmentation loss $L_{seg}$ is a standard cross-entropy loss applied between the decoder output logits and the ground-truth labels. It serves as the primary driving force for system optimization.
(b) The auxiliary segmentation loss $L_{aux}$ is applied to the output of the auxiliary segmentation head in the adaptive quantization module described in Section 4.3. It encourages this branch to learn meaningful class posterior distributions, ensuring that the utility map U(i,j) derived from entropy and class importance reflects actual semantic uncertainty.
(c) The rate constraint loss $L_{rate}=\left|\bar{b}-b_{target}\right|$, where $\bar{b}$ denotes the average quantization bit-width of the current batch and $b_{target}=4.0$ denotes the target bit-width. This term constrains the adaptive allocation mechanism to perform bit-width redistribution while keeping the global average bit-width consistent with the fixed 4-bit baseline, thereby ensuring fairness in comparison.
(d) The entropy regularization term $L_{ent}$ imposes an entropy constraint on the probability distribution over quantization levels, preventing the allocation probabilities from collapsing prematurely to a single level in the early training stage and encouraging exploration.

The weighting coefficients are set to $\lambda_{aux}=0.4$, $\lambda_{rate}=0.1$, and $\lambda_{ent}=0.05$, determined by jointly examining the distribution of quantization levels and segmentation performance on the validation set.

To coordinate the difference in optimization speed between the pretrained encoder and the other modules trained from scratch, this paper adopts a layer-wise learning-rate strategy. The encoder learning rate is set to $6\times 10^{-5}$, while the learning rate for the remaining modules, including $\theta_{adapter}$, $\theta_{quant}$, $\theta_{dec}$, and the auxiliary segmentation head, is set to $3\times 10^{-4}$. The AdamW optimizer [28] is used, together with a polynomial learning-rate decay scheduler with a decay exponent of 1.0.

In addition, the temperature parameter τ in the Hard Gumbel-Softmax module described in Section 4.3 follows an exponential annealing schedule. It is initialized to $\tau_{0}=5.0$ and decays epoch by epoch to $\tau_{final}=0.5$. The high-temperature stage allows the allocation decisions to retain stronger stochasticity for sufficient exploration of the allocation space, whereas the low-temperature stage gradually drives the allocation strategy toward deterministic hard decisions, aligning it with the argmax behavior used during inference.

## 5. Experiments and Analysis

### 5.1. Experimental Settings and Evaluation Baselines

To comprehensively evaluate the performance of the proposed end-to-end semantic communication system, we conduct systematic experiments on the Cityscapes dataset [24]. Cityscapes is a representative benchmark for urban scene understanding in autonomous driving, containing 2975 training images and 500 validation images. In the experiments, all input images are uniformly resized to 512×1024 to accommodate hardware constraints and improve training efficiency. The evaluation metric is mean Intersection over Union (mIoU), computed over the standard 19 semantic classes of Cityscapes.

#### 5.1.1. Training Strategy and Hardware Environment

We implement the system in PyTorch 2.7.0 with CUDA 12.8. All models are trained and evaluated on a single NVIDIA RTX 5090 GPU with 32 GB memory. The entire end-to-end network is jointly trained for 100 epochs, with the batch size set to 12. The AdamW optimizer is used together with a layer-wise learning-rate strategy. To avoid disrupting the representation learned by the pretrained visual backbone, the learning rate of the SegFormer encoder backbone is conservatively set to $6\;\times \;10^{-5}$, while the learning rate of the remaining newly introduced components, including the adaptive quantization module, the channel adapter, and the feature decoder, is set to $3\;\times \;10^{-4}$. For the AWGN and Rayleigh channels, we train two independent models, each trained only under its corresponding channel type. During training, the physical-channel SNR is randomly sampled from the set {−6,0,6,12,18}dB at each forward pass, enabling the model to generalize across different SNR conditions under the same channel type. During evaluation, the system is tested independently at the specified SNR anchor points. Within the same channel type and the trained SNR range, the model does not need to be retrained for each SNR value. If the channel family, mobility pattern, or fading statistics change substantially, additional fine-tuning or validation would be required before deployment in safety-critical autonomous-driving scenarios.

The computational overhead of the newly introduced modules is limited compared with the SegFormer-B2 backbone. According to the implemented network structure, the SNR-conditioned channel adapter contains approximately 0.066 million trainable parameters, the auxiliary allocation branch contains approximately 0.068 million trainable parameters, and the lightweight task-oriented decoder contains approximately 1.55 million trainable parameters. The adaptive quantizer and wireless channel layer introduce no trainable parameters. As a separate implementation-level profiling check on a local PyTorch/CUDA platform with an NVIDIA RTX 4070 Laptop GPU, the non-encoder modules have small per-sample runtime: approximately 0.181 ms for the channel adapter, 0.862 ms for the allocation branch, 0.638 ms for the adaptive quantizer, 0.156 ms for the AWGN channel layer, and 0.620 ms for the decoder. These profiling values are hardware-dependent and are reported only to indicate the relative overhead of the newly introduced modules, rather than absolute deployment latency. The main computational burden is still concentrated in the semantic encoder. This design choice is consistent with the intended use case, where the transmitter extracts compact semantic features and the receiver applies a relatively lightweight task-oriented decoder. Detailed embedded-system latency evaluation is left for future deployment-oriented work, because actual latency depends on hardware, compiler, and parallelization settings.

#### 5.1.2. Baselines

To evaluate the effectiveness of the proposed system, we design the following four comparison and evaluation schemes.

(1) **Reference upper bound:** This refers to the local visual inference performance of the original SegFormer model without compression or channel transmission distortion. It serves as the theoretical mIoU upper bound achievable by the system architecture.
(2) **Traditional Separate:** This baseline follows the classical “source coding + channel transmission” paradigm. Specifically, the input image is first compressed using JPEG [29]. A digital-link abstraction based on theoretical bit error rate (BER) is then used to simulate the transmission distortion of the compressed bitstream over wireless channels. According to the theoretical BER of QPSK modulation under AWGN or Rayleigh channels [30], together with an equivalent error-correction gain introduced by a 1/2-rate channel code, random bit errors are injected into the JPEG bitstream. If the corrupted bitstream cannot be correctly parsed by the JPEG decoder, the sample is counted as a decoding failure. This baseline is used to characterize the cliff effect faced by conventional separate image transmission over error-prone channels.
(3) **Proposed-Fixed:** This scheme uses the proposed end-to-end architecture but removes the adaptive allocation module. Uniform discrete quantization, such as fixed 4-bit quantization, is applied globally. This setting is used to evaluate the noise robustness of the end-to-end joint coding architecture compared with the conventional separate scheme.
(4) **Proposed-Adaptive:** This scheme deploys the complete semantic-aware adaptive quantization mechanism. Under the constraint that the average quantization bit-width is comparable to that of the fixed 4-bit scheme, it is used to examine the behavior of non-uniform resource allocation based on semantic importance under complex channel conditions.

For bandwidth measurement, since the proposed system is based on analog feature transmission, namely analog DeepJSCC, the quantized features are transmitted through the channel as continuous-amplitude values, and no bit-to-symbol mapping is performed at the channel layer. Therefore, this paper uses the channel bandwidth ratio (CBR) to measure the transmission overhead [4,5], defined as the number of channel symbols per source pixel. The proposed system transmits 512×16×32 feature values for each image, corresponding to CBR=0.5. For the precision of the adaptive quantization mechanism itself, this paper uses average latent bit-width, defined as the average assigned bit-width over all spatial feature locations. The average latent bit-width of the proposed adaptive scheme is approximately 4.0, matching the fixed 4-bit baseline to compare bit-width allocation under the same average latent precision.

It should be noted that analog DeepJSCC and conventional digital image transmission use different bandwidth measurement systems. CBR measures channel uses per source pixel, whereas the JPEG baseline is naturally described by a compressed bitstream length together with an assumed digital modulation/coding model. Therefore, the comparison with the JPEG + BER baseline is not intended to be a bit-level capacity-equivalent comparison between two fully optimized physical-layer systems. Instead, it provides a task-oriented robustness reference under a comparable low-bandwidth transmission regime. In our validation set, the JPEG baseline with Q=50 produces an average bitstream length of approximately 1.137 bpp, or 596.1 kbits per 512×1024 image. By contrast, under the CBR =0.5 channel-use budget of the proposed system, the real-valued AWGN capacity $C_{\mathrm{AWGN}}=\frac{1}{2}\log_{2}\left(1+\mathrm{SNR}\right)$ corresponds to only about 42.4 kbits per image at SNR=−6dB and 131.1 kbits per image at SNR=0dB. This back-of-the-envelope comparison further indicates that reliable digital recovery of a JPEG bitstream is extremely difficult in the low-SNR region. The proposed semantic system does not violate this limit because it does not attempt to recover an error-free bitstream or reconstruct the image; instead, it learns a task-oriented analog feature mapping whose output is directly evaluated by segmentation mIoU.

### 5.2. Main Results: End-to-End System vs. Conventional Scheme

**Figure 2:**
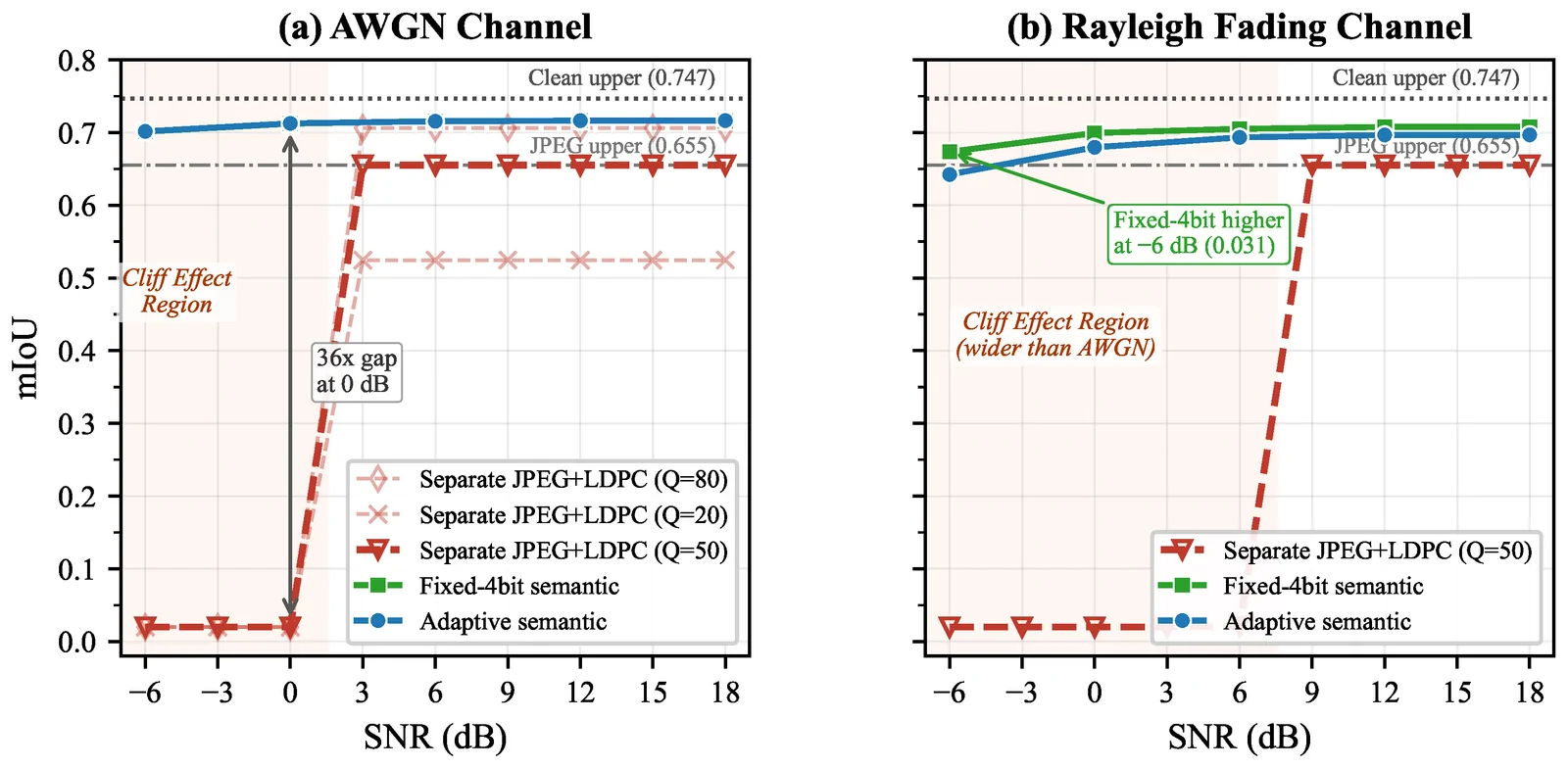
mIoU performance under the AWGN and Rayleigh fading channels.

Table 1 and Table 2, together with Figure 2, present the mIoU performance of different schemes under different SNR conditions over the AWGN and Rayleigh channels.

From Table 1 and Figure 2a, it can be observed that, under the AWGN channel, when SNR is at the low-SNR failure region, the mIoU of the conventional separate scheme drops sharply to 0.02. At this point, the JPEG bitstream cannot be correctly decoded due to channel bit errors, exposing the typical cliff effect of digital communication. In contrast, the proposed end-to-end scheme still maintains an mIoU of about 0.70 under the extremely harsh condition of SNR=−6dB. This comparison demonstrates the robustness and graceful degradation of the end-to-end DeepJSCC architecture under harsh channel conditions. In the high-SNR region, the conventional scheme approaches its JPEG performance upper bound of about 0.655, while the proposed scheme approaches about 0.716, and both enter a saturated regime. This indicates that when the channel condition is sufficient to support reliable transmission, the system bottleneck shifts from channel noise to source-side compression and feature extraction limits. It should be noted that the proposed scheme and the conventional scheme adopt different bandwidth measurement systems, namely CBR and bits per pixel (bpp), respectively. As discussed in Section 5.1, these two metrics are not directly convertible, and the comparison should be understood as a task-oriented robustness reference within a comparable low-bandwidth regime rather than as a strict physical-layer capacity equivalence. The ablation results further show that, even when the average quantization bit-width is reduced to 2-bit, the proposed system still clearly outperforms the conventional scheme under low-SNR conditions. After confirming the overall advantage of the end-to-end architecture, we further compare different bit-width allocation mechanisms within the system. Under the AWGN channel, the global mIoU difference between Proposed-Adaptive and Proposed-Fixed is small, less than 0.001. This suggests that the adaptive quantization mechanism can maintain global segmentation accuracy comparable to fixed-bit quantization under the same average latent bit-width.

Under the Rayleigh channel, as shown in Table 2 and Figure 2b, the critical SNR threshold at which the conventional scheme suffers from the cliff effect shifts markedly from the 0–3 dB range under AWGN to the 6–9 dB range. This indicates that severe channel fading forces the conventional separate architecture to require a higher SNR margin to maintain a basic communication link. In contrast, the proposed scheme still maintains continuous graceful degradation over the entire SNR range. Its advantage is particularly clear in the low-SNR region of SNR⩽6dB. For example, at SNR=0dB, the proposed scheme maintains an mIoU of about 0.68, whereas the conventional scheme has completely failed.

However, it is worth noting that, under the Rayleigh channel, Proposed-Adaptive shows lower global mIoU than Proposed-Fixed. For example, at SNR=−6dB, the two schemes obtain 0.6423 and 0.6734, respectively. This behavior differs from the AWGN case, where the two schemes show similar performance. It suggests that the adaptive quantization mechanism may involve a certain underlying mismatch when facing complex fading. The possible mechanism behind this phenomenon is analyzed in detail in Section 5.3.

### 5.3. Analysis of Semantic-Channel Mismatch

It is worth noting that, under Rayleigh fading, the global mIoU of Proposed-Adaptive is lower than that of Proposed-Fixed at all SNR levels. For example, at SNR=−6dB, the two schemes obtain 0.6423 and 0.6734, respectively. This occurs even though both schemes are evaluated under the same target bit-width budget. This section analyzes the possible mechanism behind this phenomenon.

We refer to this phenomenon as semantic-channel mismatch in joint source-channel coding. This phenomenon may not be simply attributed to a failure of the transmitter-side allocation mechanism itself. Instead, it may reflect a potential tension between the “importance” of high-dimensional semantic features and their “anti-fading robustness” at the physical layer. Specifically, at the transmitter, the adaptive allocator tends to assign high-precision 6-bit resources to regions with high local information entropy, which usually correspond to object boundaries and complex small objects. However, from the perspective of the relationship between quantization interval and channel noise, a higher quantization precision leads to smaller quantization intervals, making the numerical differences preserved by fine quantization more likely to be masked by channel perturbations of comparable magnitude. Under the AWGN channel, the statistical behavior of additive Gaussian noise is relatively mild, and the feature-value differences preserved by 6-bit quantization may remain sufficiently distinguishable from the noise amplitude. Under Rayleigh fading, however, the random amplitude variation of the fading coefficient *h* acts on the transmitted signal through a multiplicative effect. In deep fading events where |h| is small, the equivalent noise variance after zero-forcing (ZF) equalization at the receiver is markedly amplified by $1/{\left|h\right|}^{2}$ [30], causing severe perturbations to the numerical differences retained by fine quantization.

It should be noted that, in the current experimental results, the performance degradation of Proposed-Adaptive relative to Fixed-4bit may be related to the introduction of a certain proportion of 6-bit units. The finer quantization intervals introduced by higher bit-widths may be more vulnerable to perturbations under Rayleigh fading and equalization-induced noise amplification, and therefore may not be stably converted into semantic protection gains. This explanation remains a mechanism analysis based on experimental observations, rather than a general conclusion for all bit-width configurations.

This physical fading effect leads to a noteworthy phenomenon: the adaptive mechanism tends to allocate higher quantization precision, such as 6-bit, to core semantic regions, but these high-precision features may also be more vulnerable to perturbation and quantization-level shifts when passing through the fading channel. In contrast, background regions assigned lower bit-widths, such as 2-bit, are more coarsely quantized, but their larger quantization intervals may provide relatively stronger anti-fading robustness. This mismatch, where high-precision regions may have weaker perturbation resistance, may be one reason why the system under severe fading achieves lower global mIoU than the uniformly distributed fixed 4-bit strategy.

This observation suggests that, under fading channels, relying solely on transmitter-side high-precision bit-width bias may be insufficient to protect critical semantics. A potential future direction is that task-oriented semantic communication may need not only to allocate more source-side resources to important semantics, but also to provide corresponding error protection for these features at the channel side, thereby mitigating the mismatch between semantic importance and channel robustness. This provides a useful reference for future semantic communication system design over fading channels.

### 5.4. Ablation Study

To evaluate the role of each key component in the proposed end-to-end semantic communication system, this section mainly conducts a complete ablation study under the AWGN channel and further combines partial comparisons under the Rayleigh fading channel to analyze the limitations of the model in fading environments. The ablated components include the channel adapter, the auxiliary loss, and different quantization bit-width strategies, including fixed 2-bit, 4-bit, 6-bit, and adaptive quantization. The results are reported in Table 1, Table 2 and Table 3. Complete ablation results are provided under AWGN, while partial comparisons under Rayleigh are included to analyze the limitations of the model in fading environments.

**Role of the Channel Adapter:** We compare the system performance with and without the channel adapter. The purpose of retaining this module in the present system is not to claim that it already provides stable performance gains, but to explicitly test whether a lightweight SNR-conditioned channel-wise modulation can help bridge the gap between semantic features and channel states. Under the AWGN channel, removing this module has only a small impact on mIoU, with the maximum difference across SNR levels being only 0.0011. This indicates that the current channel adapter provides limited gain in the additive Gaussian noise environment. Under Rayleigh fading, comparing the complete Proposed-Adaptive model with the NoAdapter variant, for example 0.6423 versus 0.6489 at SNR=−6dB, shows that the adapter does not bring stable positive gains under this configuration. We also inspect the learned SNR-conditioned channel gates. For the AWGN-trained model, the mean gate value changes from approximately 0.914 at SNR=−6dB to 0.864 at SNR=18dB; for the Rayleigh-trained model, it changes from approximately 0.927 to 0.801 over the same SNR range. This confirms that the adapter does learn an SNR-dependent recalibration pattern, but the learned channel-wise reweighting is not sufficient to produce stable segmentation gains under the current architecture. This result indicates that the coupling between the current form of the channel adapter and the adaptive quantization mechanism is still insufficient: simply reweighting feature channels according to SNR is not enough to compensate for the degradation of high-precision quantized features caused by fading channels. Therefore, this module is better viewed as a diagnostic design choice and a limitation-revealing component rather than a mature performance-improving component. Designing a channel-adaptive module that is more cooperative with the quantization mechanism is therefore a direction worth further investigation. It should also be noted that removing the channel adapter changes the feature distribution received by the subsequent allocator and may be accompanied by slight changes in average latent bit-width. Therefore, this ablation result is more suitable for understanding module-coupling effects and should not be interpreted as a strict single-factor performance proof under a fixed-rate setting.

**Semantic Guidance Value of the Auxiliary Loss:** Under the AWGN channel, while keeping the rest of the system unchanged, we further evaluate the effect of removing the auxiliary loss, referred to as NoAux. The results show that, under the extremely harsh condition of SNR=−6dB, removing this constraint reduces the system mIoU from 0.7016 to 0.6940. This indicates that relying solely on the segmentation loss at the final decoder output is still insufficient. The auxiliary loss directly applies segmentation supervision at the encoder output, shortening the gradient propagation path. Without this constraint, the segmentation supervision signal must propagate from the decoder output through the channel, quantizer, and adapter before reaching the encoder. Such an excessively long backpropagation path may severely dilute the gradient signal, making it difficult for the encoder to effectively preserve the core spatial structures relevant to the segmentation task.

**Source-Channel Trade-off and Bit-Width Allocation:** To investigate the effect of bit-width allocation on end-to-end performance, we compare fixed-bit-width schemes, namely 2-bit, 4-bit, and 6-bit, with the proposed adaptive quantization module. The experimental results reveal a noteworthy phenomenon: across all SNR levels, Fixed-4bit not only consistently outperforms Fixed-2bit in global performance, but also outperforms Fixed-6bit despite the latter using higher precision. At SNR=6dB, Fixed-4bit achieves an mIoU of 0.7158, whereas Fixed-6bit achieves only 0.7099, even though it uses a higher quantization bit-width. This phenomenon indicates that, in noisy wireless channels, increasing quantization precision reduces the quantization interval, thereby decreasing the relative margin between the numerical differences preserved by fine quantization and channel noise of comparable magnitude. As a result, the perturbation effect of noise on finely quantized values can be amplified, offsetting the feature-preservation benefit brought by higher quantization precision. This suggests that, in noisy channel environments, there exists a suitable quantization precision matched to the channel condition. Simply increasing quantization precision not only increases bandwidth overhead, but may also degrade system performance.

**Fine-Grained Feature Analysis and Limitations of the Adaptive Mechanism:** Although the fixed 4-bit scheme shows the best source-channel trade-off in terms of global average metrics, the global mean can hide fine-grained differences across semantic categories. To examine the behavior of different bit-widths on specific classes, we compare the per-class IoU of fixed 2-bit, 4-bit, and 6-bit settings. As shown in Table 3, although fixed 6-bit is worse than 4-bit in terms of global performance, it still achieves the best accuracy on some fine-grained categories, such as traffic light, 0.5334 versus 0.5266, traffic sign, 0.6201 versus 0.6185, and classes such as person and rider. This suggests that higher quantization precision has some potential for preserving local semantic details of specific critical categories. However, such category-level gains are not stable and are offset by degradation on categories such as truck, bus, and car, eventually leading to lower global mIoU. Therefore, the experimental results do not support the simple conclusion that “higher bit-width is necessarily better.” Instead, they indicate that semantic resource allocation needs to jointly consider class importance, spatial granularity, and channel robustness.

We further observe that adaptive quantization, with an average latent bit-width of approximately 4.0, and Fixed-4bit, with bit-width equal to 4.0, show only small differences in both global mIoU and per-class performance under the same bit-width budget. This indicates that, at the current spatial resolution of 16×32, the allocator has not yet converted differentiated allocation into measurable performance gains. The main reason for this limitation is that the allocation granularity is too coarse: each allocation unit corresponds to a 32×32 pixel region in the original image. Small objects are often mixed with large background regions within the same allocation block, causing semantic aliasing and making it difficult for the allocator to realize pixel-level precise resource bias. Exploring allocation strategies at higher spatial resolutions is a direction worth studying in future work.

### 5.5. Visualization Analysis

**Figure 3:**
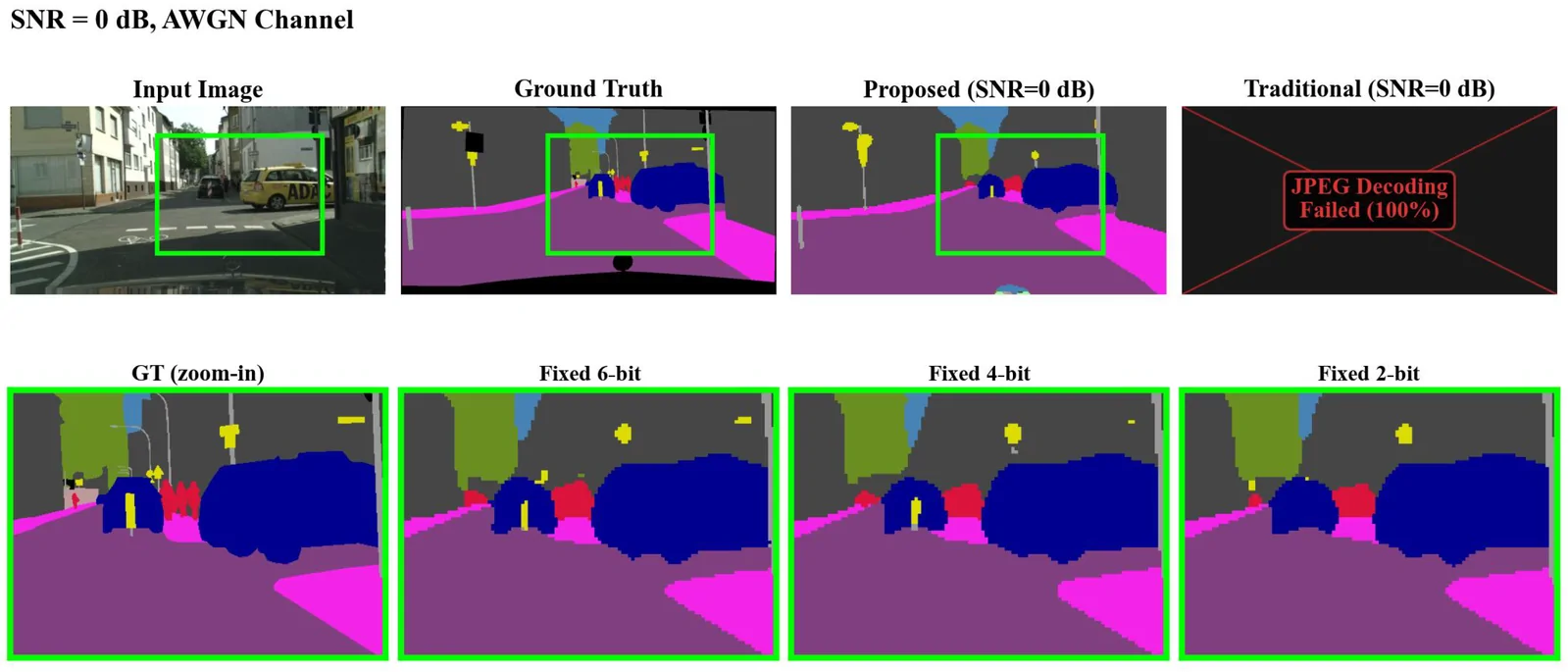
Visual comparison of segmentation results under the AWGN channel at SNR=0dB. Segmentation colors follow the standard Cityscapes class palette, and green boxes indicate the region of interest enlarged in the lower row.

The upper row of Figure 3 presents the overall segmentation comparison of different schemes under the AWGN channel at SNR=0dB. The conventional separate scheme fails to produce any valid segmentation output because JPEG decoding completely fails, with a failure rate of 100%. In contrast, under the same extreme channel condition, the proposed end-to-end scheme can still correctly identify key targets such as road, vehicles, pedestrians, and traffic facilities, remaining largely consistent with the ground-truth segmentation map and preserving key structures. This visually supports the quantitative results in Section 5.2, which show that the end-to-end architecture can suppress the cliff effect.

The lower row of Figure 3 further compares the segmentation details of the three fixed-bit-width schemes within the region of interest under the proposed architecture. Fixed-6bit produces the sharpest segmentation results around large vehicle boundaries, pedestrian contours, and small traffic-light targets, with the highest boundary alignment to the ground truth. Fixed-4bit is close to Fixed-6bit in terms of vehicle body structure, but shows slight boundary smoothing around distant small objects and thin structures. Fixed-2bit exhibits clear blocky stair-step artifacts near the lower vehicle boundary, while distant traffic-light targets and other small objects show severe degradation or confusion. This visual gradient is consistent with the per-class IoU results in Table 3 for some fine-grained categories: higher quantization precision helps preserve local semantic details of certain classes. However, it should be emphasized that this local advantage of Fixed-6bit does not translate into a global mIoU advantage. This also suggests that concentrating higher precision on visually sensitive regions may have some potential in principle, but whether it can be converted into segmentation gains for critical categories without increasing the overall overhead still depends on the allocation granularity and channel conditions.

As discussed in Section 5.4, under the current 16×32 allocation granularity, the adaptive mechanism has not yet fully realized this potential. The visual comparison in this section only illustrates the precision gradient under fixed-bit-width schemes, providing intuitive motivation for future exploration of finer-grained allocation strategies on higher-resolution features.

## 6. Conclusions

This paper proposes an end-to-end semantic communication system based on the deep joint source-channel coding framework to meet the demand for low-latency and robust wireless transmission in autonomous-driving semantic segmentation tasks. The system consists of a SegFormer-B2 semantic encoder, an SNR-conditioned feature modulation module, an adaptive quantization allocation mechanism based on two-dimensional semantic utility, a wireless channel model, and a lightweight task-oriented decoder. Through multi-SNR joint training, the system adapts to dynamic SNR conditions.

Experiments on the Cityscapes dataset show that the proposed end-to-end semantic communication system is more robust than the conventional separate JPEG transmission baseline under low-SNR conditions. Specifically, under the AWGN channel at SNR=0dB, the conventional scheme nearly fails because the JPEG bitstream corrupted by bit errors cannot be correctly parsed, whereas the end-to-end semantic transmission system still maintains an mIoU of about 0.71. Further ablation experiments show that the relationship between quantization bit-width and semantic segmentation performance is not simply monotonic. Fixed-4bit outperforms Fixed-6bit in multiple settings, suggesting that higher quantization precision may fail to translate into global performance gains due to stronger noise sensitivity. Meanwhile, the adaptive quantization mechanism can maintain performance close to Fixed-4bit under the AWGN channel, but exposes a mismatch between semantic-importance allocation and channel robustness under Rayleigh fading. The current ChannelAdapter should therefore be interpreted as a diagnostic and limitation-revealing component whose standalone gains remain limited under the present architecture. These observations provide experimental evidence for future design of stronger channel-aware semantic resource allocation mechanisms.

This work has several limitations. First, the spatial granularity of adaptive quantization allocation is limited by the 16×32 resolution of the encoder output feature map. Each allocation unit corresponds to a 32×32 pixel region in the original image, making it difficult to realize pixel-level fine-grained allocation. When an allocation unit covers cross-class regions, the allocation mechanism cannot differentiate within the unit. This may be a structural reason why adaptive allocation does not show systematic advantages over fixed allocation in the per-class IoU analysis. Second, the Rayleigh fading model used in this paper is per-channel independent block fading, and does not model more complex practical channel characteristics such as temporal correlation, multipath propagation, and frequency selectivity. Third, the current formulation considers a single-link transmission setting. It does not yet include multi-user interference, collision-resilient random access, pilot design, scheduling, or cooperative resource allocation, all of which are important in practical V2X networks.

Future work can proceed in three directions. First, allocation mechanism design on higher-resolution features can be explored to overcome the structural limitation of the current allocation granularity. Second, the system can be extended to channel models that are closer to practical deployment conditions, such as OFDM, temporally correlated fading, and multi-user V2X scenarios. In multi-user cases, the semantic feature transmission mechanism studied here would need to be combined with scheduling, pilot/collision handling, and interference-aware resource allocation, where collision-resilient random-access mechanisms [23] may provide useful design references. Third, dynamic rate control based on image content complexity can be designed, enabling the system to adaptively adjust transmission bandwidth according to scene semantic complexity rather than being constrained by a fixed target bit-width. 

## Figures and Tables

**Table 1:** mIoU comparison under the AWGN channel.

Scheme	−6 dB	0 dB	6 dB	12 dB	18 dB
Proposed-Adaptive	0.7016	0.7126	0.7156	0.7164	0.7165
Proposed-Fixed 4-bit	0.7014	0.7124	0.7158	0.7162	0.7162
Fixed 2-bit	0.6918	0.7088	0.7126	0.7136	0.7140
Fixed 6-bit	0.6969	0.7073	0.7099	0.7106	0.7110
w/o ChannelAdapter	0.7005	0.7118	0.7149	0.7154	0.7155
w/o auxiliary loss	0.6940	0.7090	0.7130	0.7136	0.7140
Traditional JPEG + BER (Q = 50)	0.0198	0.0198	0.6552	0.6552	0.6552

**Table 2:** mIoU comparison under the Rayleigh fading channel. The table reports the Rayleigh-channel results directly used in the main discussion. All semantic transmission schemes use CBR = 0.5, and the traditional baseline uses JPEG quality factor Q = 50.

Scheme	−6 dB	0 dB	6 dB	12 dB	18 dB
Proposed-Adaptive	0.6423	0.6798	0.6934	0.6967	0.6969
Proposed-Fixed 4-bit	0.6734	0.6995	0.7052	0.7078	0.7080
w/o ChannelAdapter	0.6489	0.6859	0.6952	0.6989	0.7001
Traditional JPEG + BER (Q = 50)	0.0198	0.0198	0.0211	0.6552	0.6552

**Table 3:** Per-class IoU comparison under the AWGN channel at SNR=6dB.

Class	Adaptive	Fixed 2-bit	Fixed 4-bit	Fixed 6-bit
Road	0.9762	0.9755	0.9761	0.9762
Sidewalk	0.8155	0.8135	0.8155	0.8142
Building	0.8977	0.8964	0.8978	0.8982
Wall	0.6116	0.5990	0.6125	0.6055
Fence	0.5407	0.5335	0.5459	0.5423
Pole	0.4348	0.4312	0.4409	0.4360
Traffic Light	0.5288	0.5185	0.5266	0.5334
Traffic Sign	0.6193	0.6110	0.6185	0.6201
Vegetation	0.8926	0.8909	0.8926	0.8930
Terrain	0.6205	0.6252	0.6270	0.6207
Sky	0.9059	0.9059	0.9052	0.9071
Person	0.6927	0.6856	0.6914	0.6945
Rider	0.5124	0.5020	0.5066	0.5073
Car	0.9107	0.9089	0.9134	0.9100
Truck	0.7913	0.7967	0.7728	0.6826
Bus	0.8261	0.8318	0.8304	0.8207
Train	0.7729	0.7826	0.7722	0.7797
Motorcycle	0.5831	0.5748	0.5882	0.5805
Bicycle	0.6628	0.6565	0.6667	0.6662
mIoU	0.7156	0.7126	0.7158	0.7099

## Data Availability

Publicly available datasets were analyzed in this study. The Cityscapes dataset is available at https://www.cityscapes-dataset.com/. All data supporting the findings of this study are included in the article.
